# Supplementary material for: A systematic evaluation of highly variable gene selection methods for single-cell RNA-sequencing
Source: Genome Biol. 2025 Dec 11;26:424. doi: 10.1186/s13059-025-03887-x (PMC12699822; doi:10.1186/s13059-025-03887-x)
Supplement: Supplementary file 1 — Additional file 1: Supplementary Figures. Figs. S1-S22 [file 13059_2025_3887_MOESM1_ESM.pdf]

# Additional File 1: Supplementary Figures

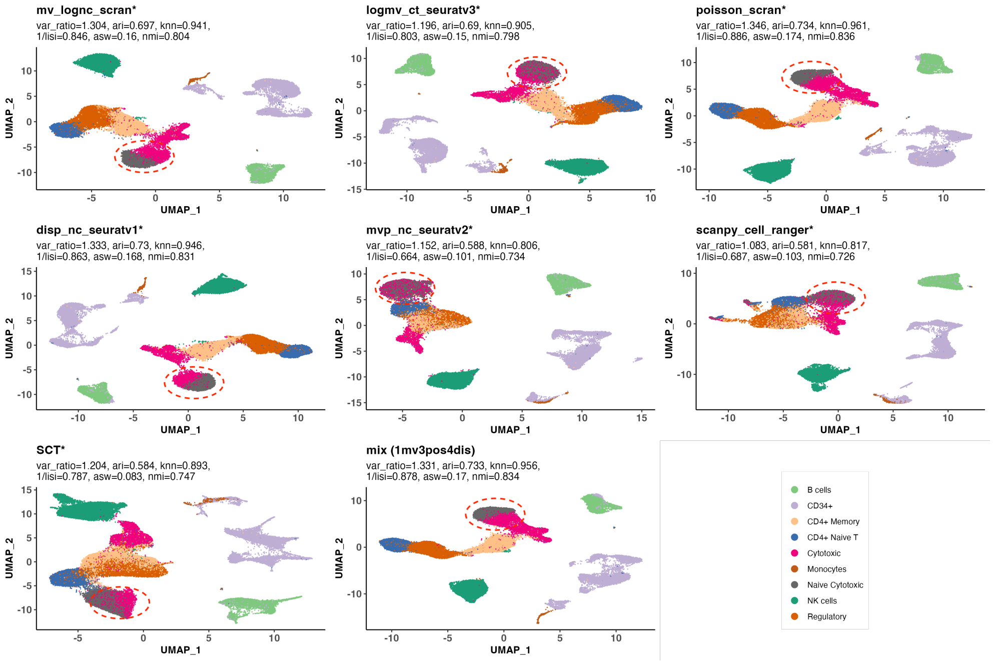


**Fig. S1**: A comparison of seven existing methods and mixHVG (default) in the zheng_pbmc dataset. Each subplot depicts a UMAP generated using scRNA-seq data and HVGs selected by a specific method. The color indicates true cell type labels obtained from cell sorting. The plot includes seven publicly available methods (denoted by *) and the default hybrid method of mixHVG (mv_lognc_scran(1mv) + poisson_scran(3pos) + disp_nc_seuratv1(4dis)). The evaluation criteria values are displayed for each method. Cell display orders are shuffled to prevent overlap between different cell types.

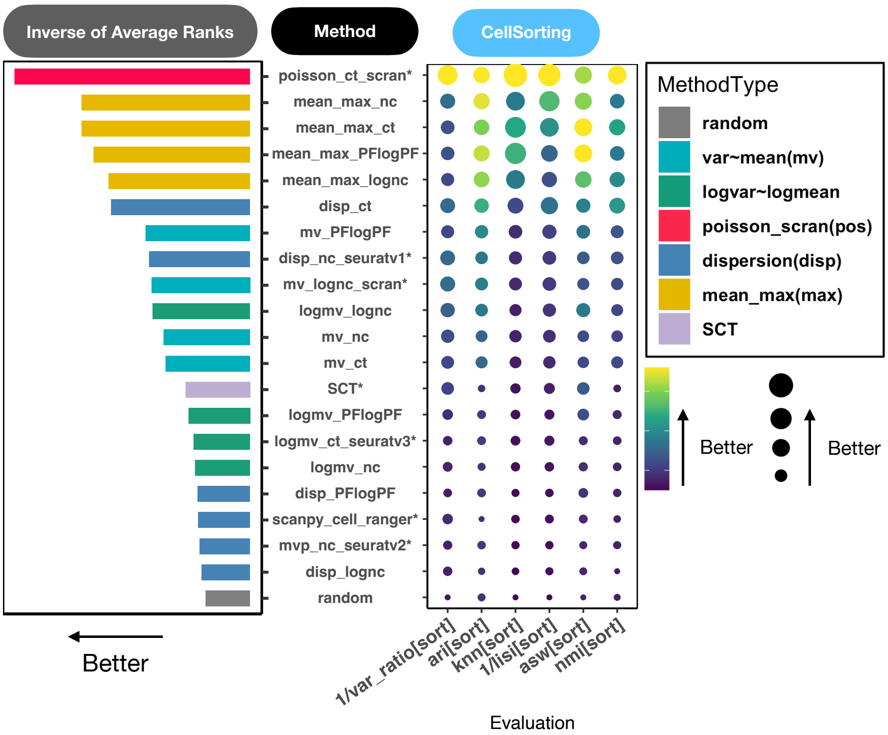


**Fig. S2**: Evaluation of baseline methods using cell sorting datasets, with methods displayed in descending order of overall performance. Each row represents a method, and each column represents an evaluation criterion. For each method and criterion, the average performance across 7 cell sorting datasets is shown in a balloon plot. Methods are ranked based on each criterion. The bar plot on the left displays the average rank across the 6 criteria.

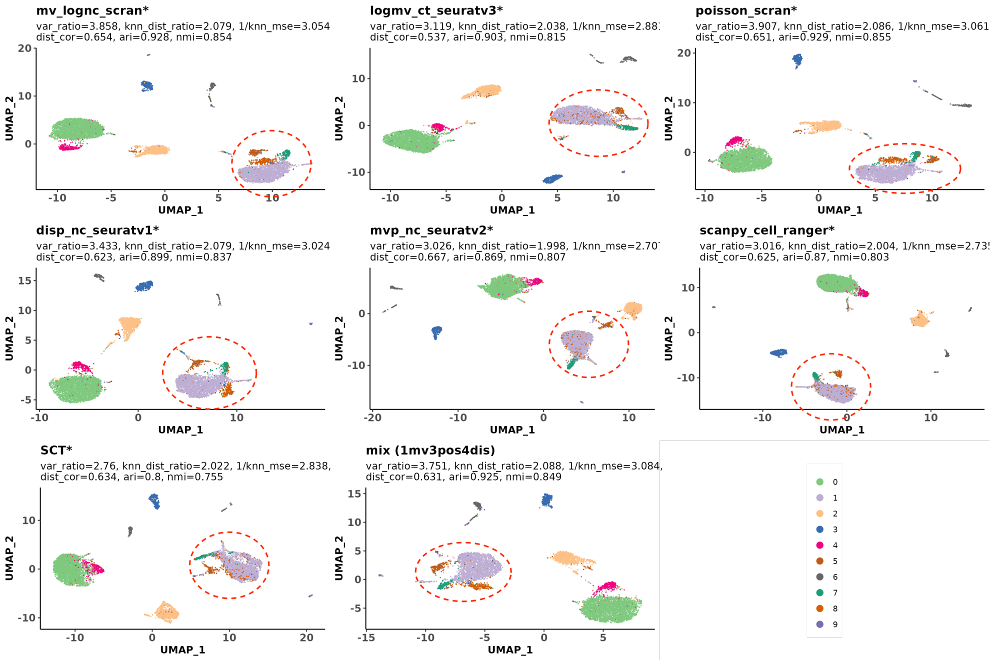


**Fig. S3**: A comparison of seven existing methods and mixHVG (default) in the cbmc8k_cite dataset. The cells are colored by the clustering results using ADT with Louvain clustering (resolution 0.2). The plot includes 7 publicly available methods (with *) and the default hybrid method of mixHVG (mv_lognc_scran(1mv) + poisson_scran(3pos) + disp_nc_seuratv1(4dis)). The values of all criteria are marked for each method. The cells are all shuffled to avoid the case where one cell type are totally covered by the other.

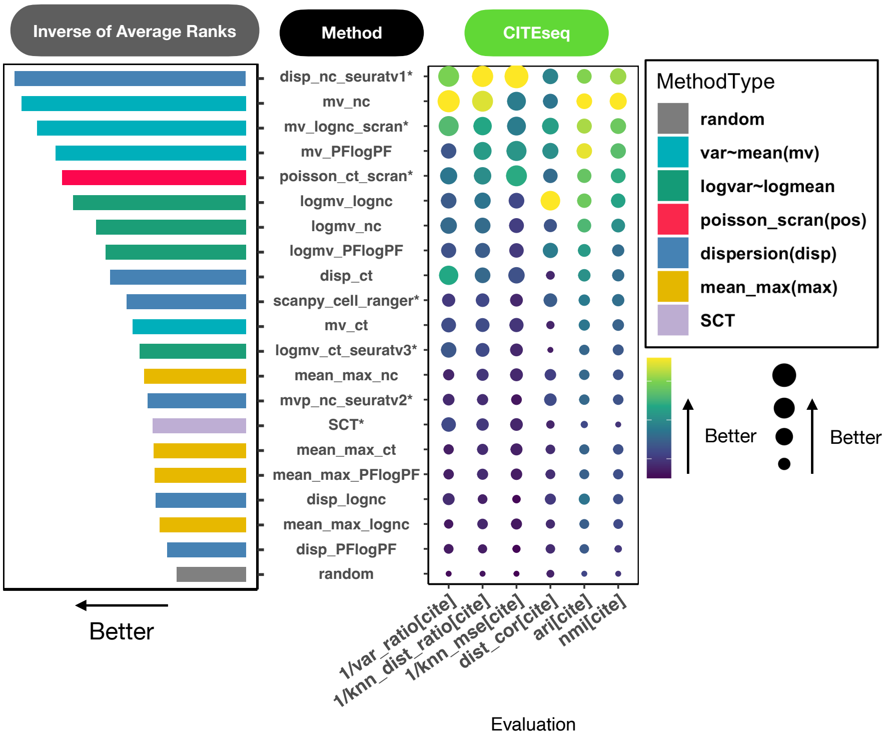


**Fig. S4**: Evaluation of baseline methods using CITE-seq, with methods displayed in descending order of overall performance. Performance of 21 baseline HVG methods across six evaluation criteria are shown. The balloon plot shows average performance across 7 CITE-seq datasets. Methods are ranked based on each criterion. The bar plot on the left displays the average rank across the 6 criteria.

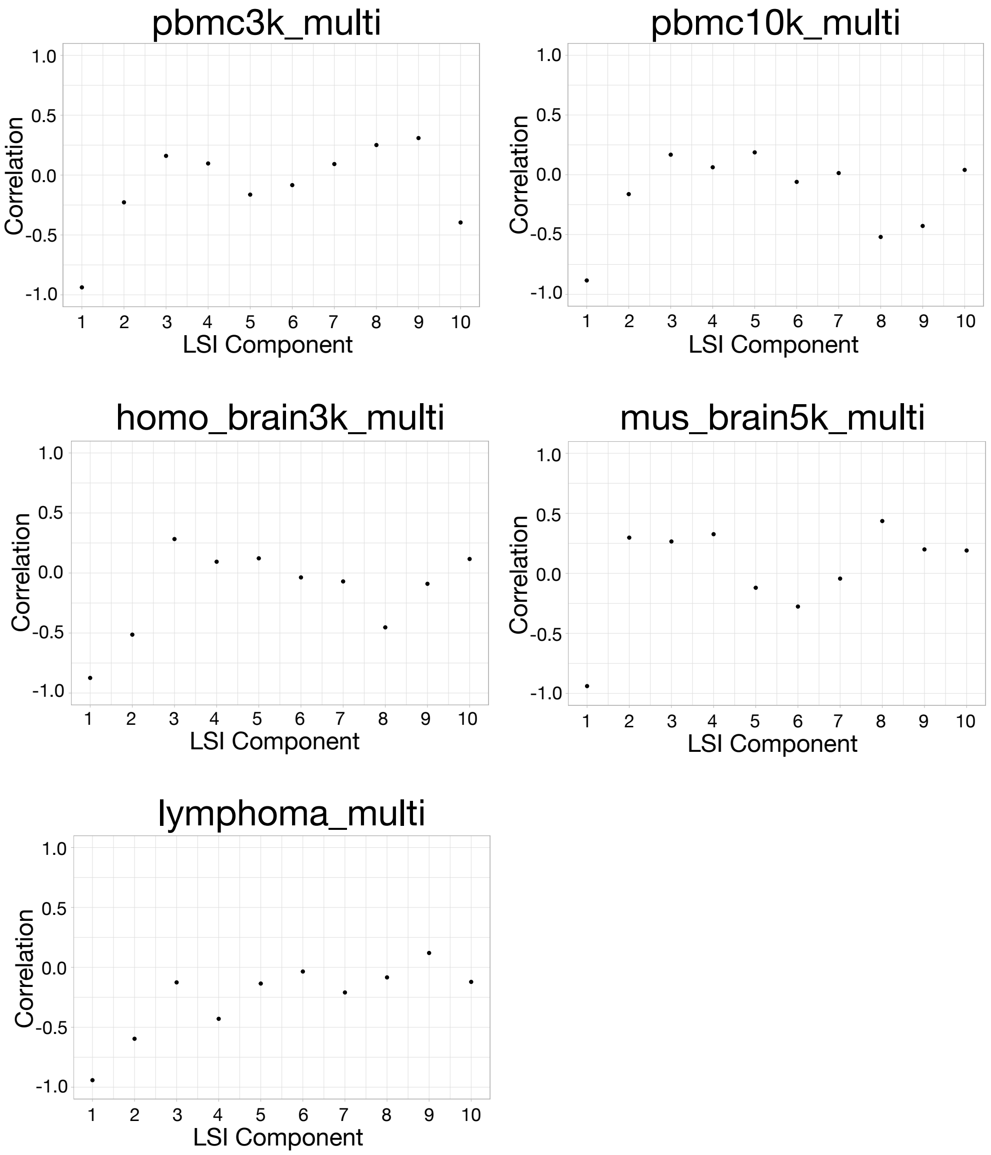


**Fig. S5**: Pearson correlation between cell's sequencing depth for scATAC (i.e., library size) and the first 10 LSI components in the single-cell multiome datasets.

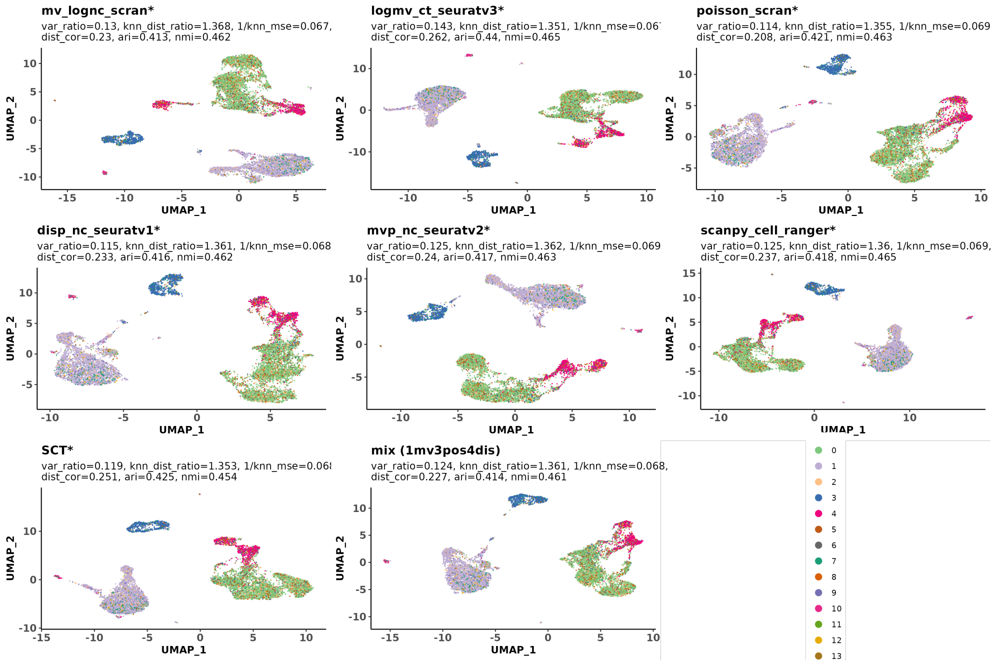


**Fig. S6**: A comparison of seven existing methods and mixHVG (default) in the pbmc10k_multi dataset. The cells are colored by the clustering results using the $2^{\text{nd}}$ to $30^{\text{th}}$ dimensional LSIs with Louvain clustering (resolution 0.2). The plot includes 7 publicly available methods (with *) and the default hybrid method of mixHVG (mv_lognc_scran(1mv) + poisson_scran(3pos) + disp_nc_seuratv1(4dis)). The values of all criteria are marked for each method. The cells are all shuffled to avoid the case where one cell type are totally covered by the other.

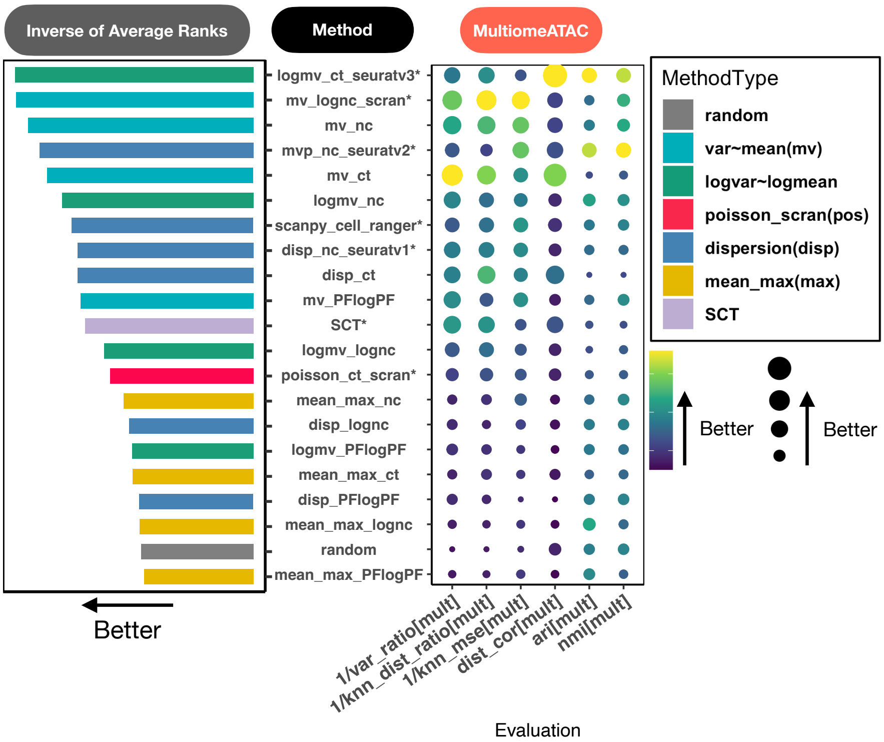


**Fig. S7**: Evaluation of baseline methods based on single-cell multiome datasets with paired scRNA-seq and scATAC-seq, with methods displayed in descending order of overall performance. Performance of 21 baseline HVG selection methods across six evaluation criteria are shown. The average performance across 5 single-cell multiome datasets is shown in a balloon plot. Methods are ranked based on each criterion. The bar plot on the left displays the average rank across the 6 criteria.

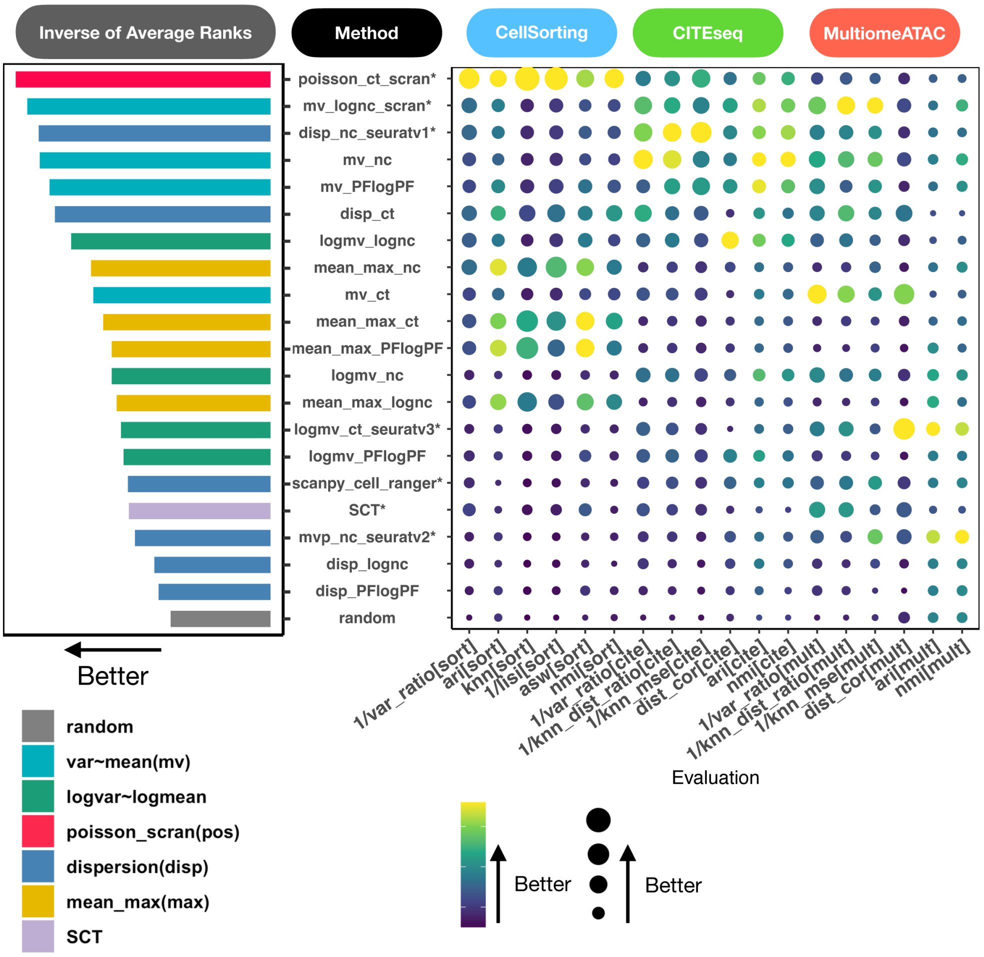


**Fig. S8**: Comparison of 21 baseline HVG selection methods across all benchmark datasets and criteria, with methods displayed in descending order of overall performance. The evaluation involves 18 criteria grouped based on benchmark data types: cell sorting, CITE-seq, and multiomeATAC. Each data type has 6 evaluation criteria. For each method and criterion, the average performance across all benchmark datasets is shown in a balloon plot. Methods are ranked based on each criterion. The bar plot on the left displays the average rank across the 18 criteria.

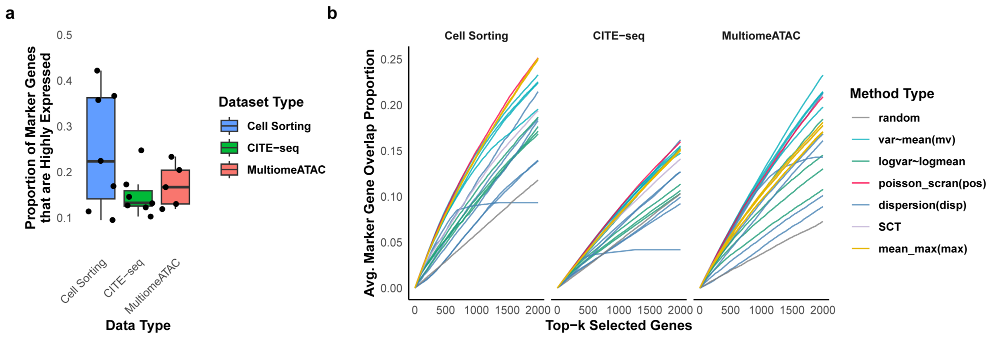


**Fig. S9**: Comparison of overlap between HVGs and cell type marker genes across three benchmark data types. (a) Boxplots show the distribution of the proportion of gold standard cell type marker genes that are highly expressed in each benchmark data type, where "highly expressed" is defined as the top 2000 genes with the highest mean expression levels. Each point represents a benchmark dataset. (b) The proportion of the top $k$ HVGs identified by each method that overlap with the gold standard cell type marker genes. Each curve represents a method, color-coded by its mean–variance adjustment approach, and depicts the method's average performance across all benchmark datasets.

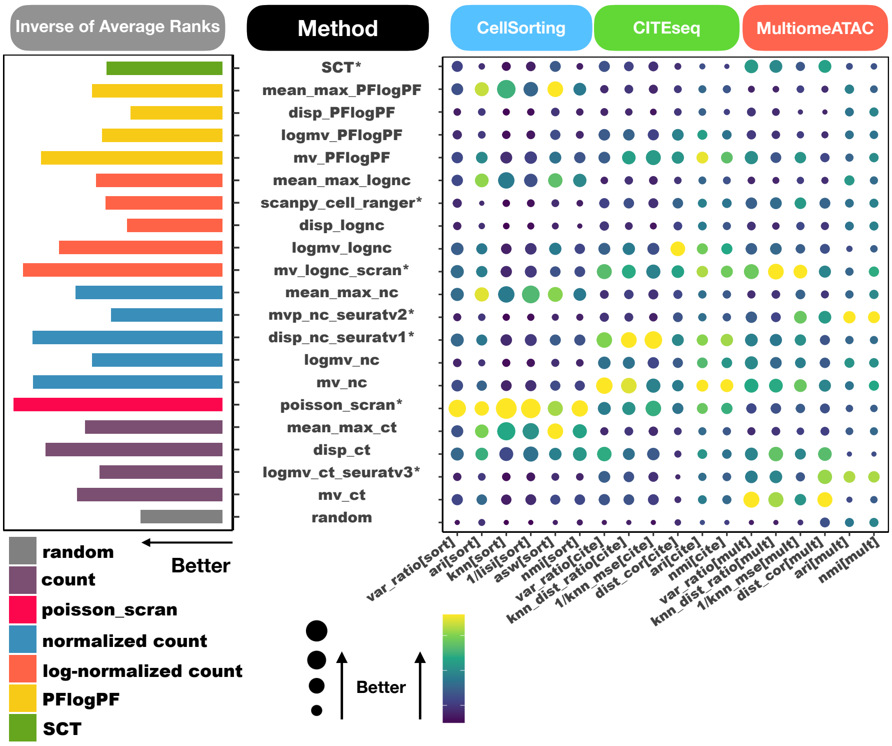


**Fig. S10**: Comparison of 21 baseline HVG selection methods across all benchmark datasets and criteria from the view of data transformation. The methods are categorized into four main groups based on input data transformation type: count, normalized count, log-normalized count, and PFlogPF. Each category is represented by a different color. The plot is a reordering of Figure 5.

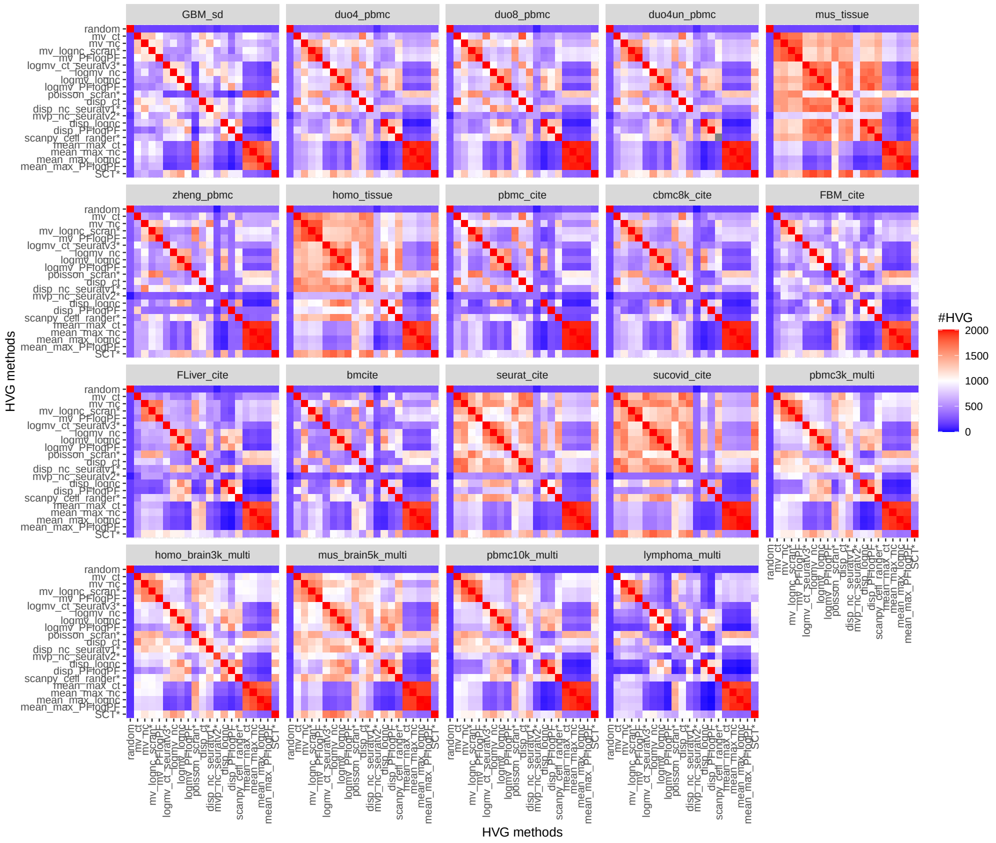

**Fig. S11**: Heatmap showing the number of overlapping genes selected by different HVG methods. Each subplot represents one dataset. Red indicates high overlap.

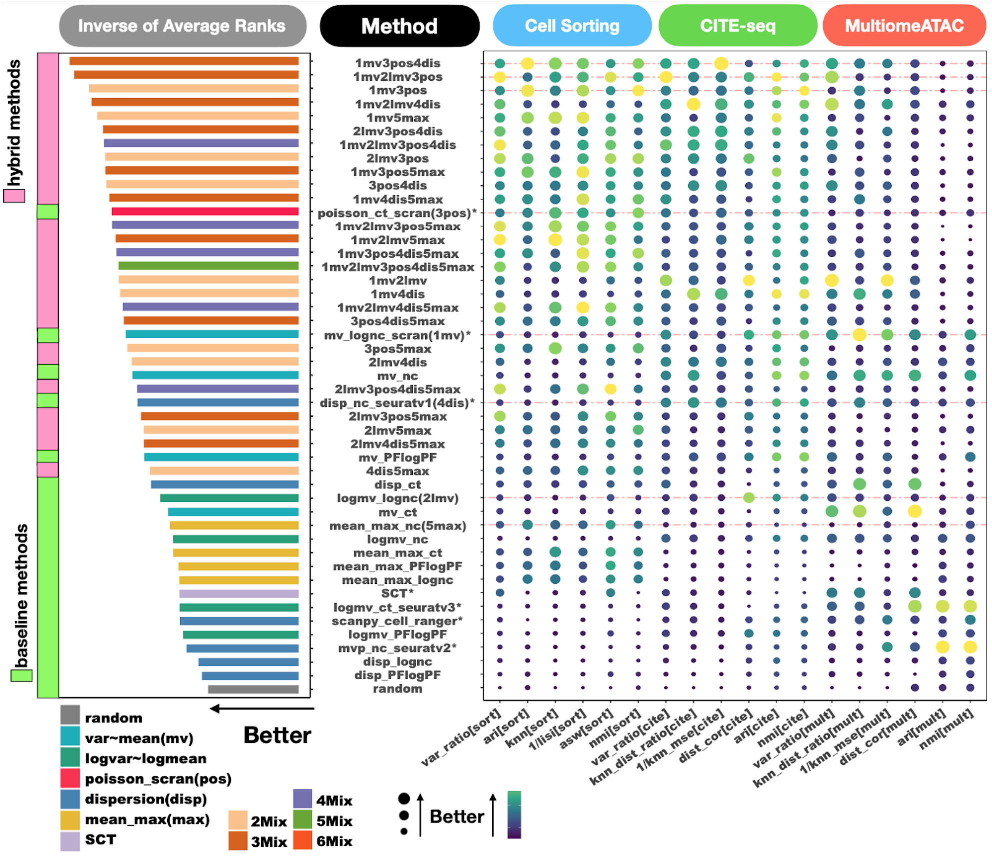


**Fig. S12**: Comparison of baseline methods and hybrid methods for selecting HVGs, with methods displayed in descending order of overall performance. The average performance across all benchmark datasets is depicted in a balloon plot for each method and evaluation criterion. Methods are ranked based on each criterion. The bar plot on the left illustrates the average rank across all 18 criteria. Hybrid methods are color-coded according to the number of baseline methods they incorporate, with each hybrid method named based on the mixture of baseline methods used. The plot is a reordering of Figure 6.

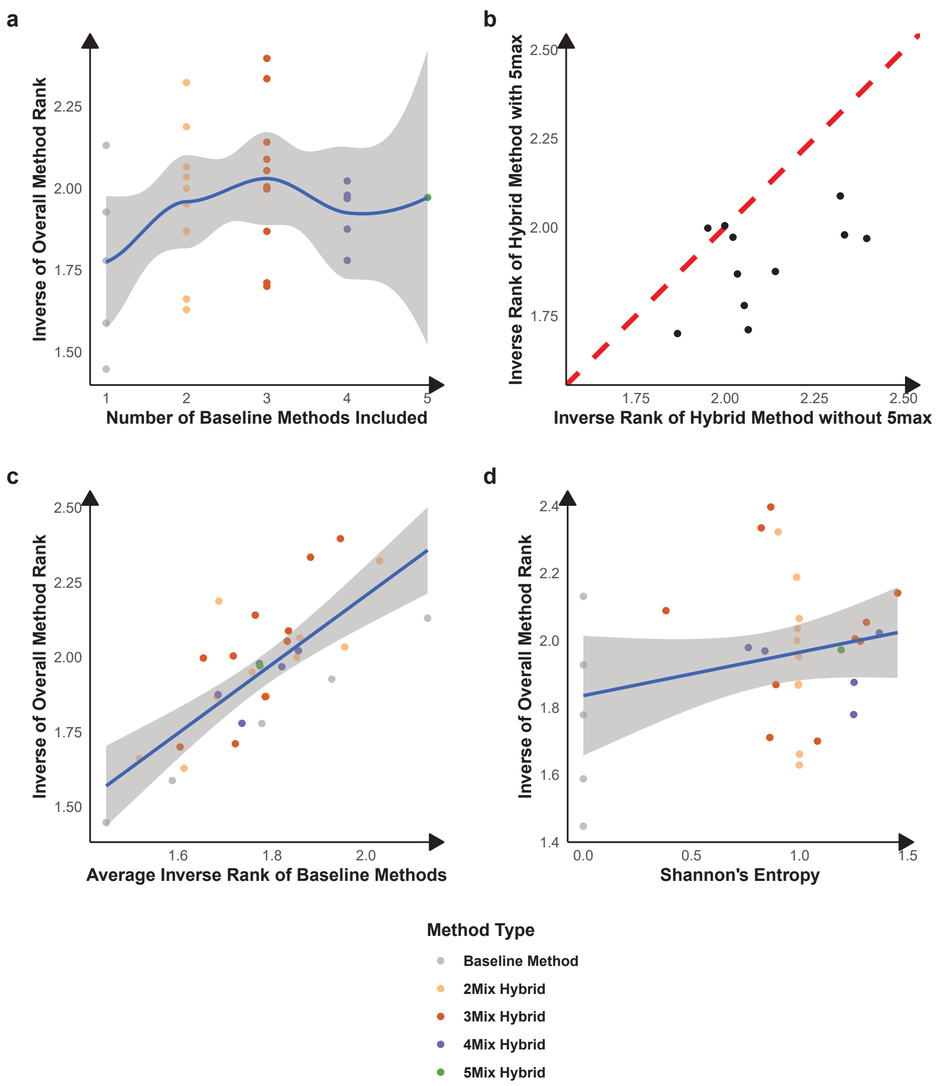


**Fig. S13**: Exploration of factors influencing the performance of hybrid HVG methods. Scatter plots show the overall performance ranks of hybrid HVG methods and the five baseline HVG methods used to construct them, plotted against **(a)** the number of baseline methods included in each method, **(c)** the average rank of the baseline methods included, and **(d)** the Shannon entropy of baseline method-specific genes within the top 2000 HVGs. The performance ranks were transformed using the inverse (max_rank/$r$, where $r$ is a method's mean rank across all evaluation criteria and max_rank is the maximum $r$ among all methods), so that larger values indicate better performance. LOESS fits (a) and linear regressions (c,d) are overlaid. **(b)** shows a scatter plot comparing the average overall performance ranks of hybrid HVG method pairs with and without 5max; points below the diagonal indicate decreased performance when 5max is included in the hybrid.

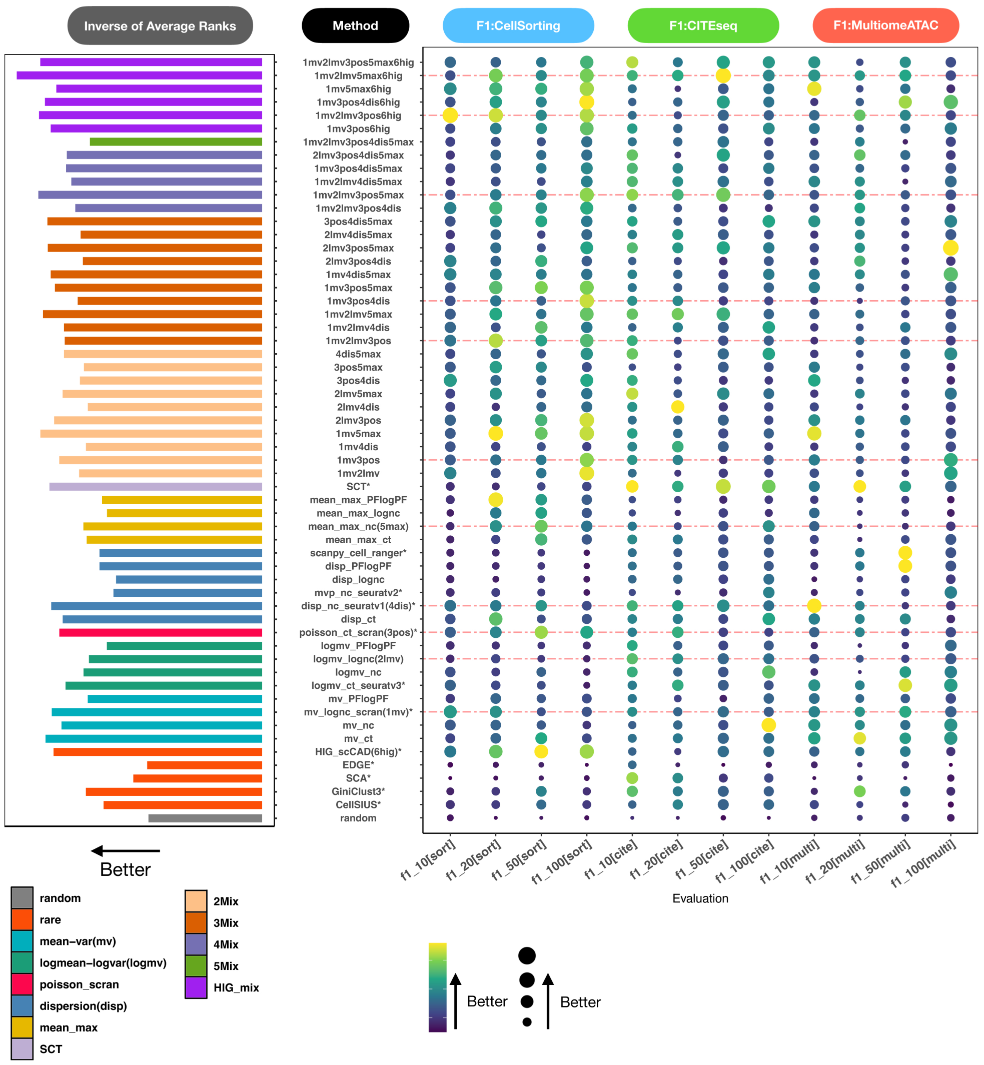


**Fig. S14**: Rare cell type identification performance using genes selected by baseline and hybrid HVG and RCTI methods, categorized by method type (HVG or RCTI), the mean-variance adjustment approach within HVG methods, and the number of baseline methods incorporated in each hybrid method. For each data type, evaluations were conducted at four rare cell type abundance levels with 10, 20, 50, or 100 cells, respectively. The balloon plots depict the average performance across all benchmark datasets for each method and abundance level, with methods ranked by the $F_1$ score. The bar plot on the left shows the average rank across all 12 evaluation settings. Hybrid HVG methods are color-coded by the number of baseline methods they incorporate, with each hybrid named according to its combination of baseline methods. Hybrids combining HVG and RCTI methods are shown in purple. In the balloon plot, the top three methods based on RCTI performance, the top three methods from the HVG benchmark in Figure 6 along with the baseline methods used to construct them are highlighted with red dashed lines.

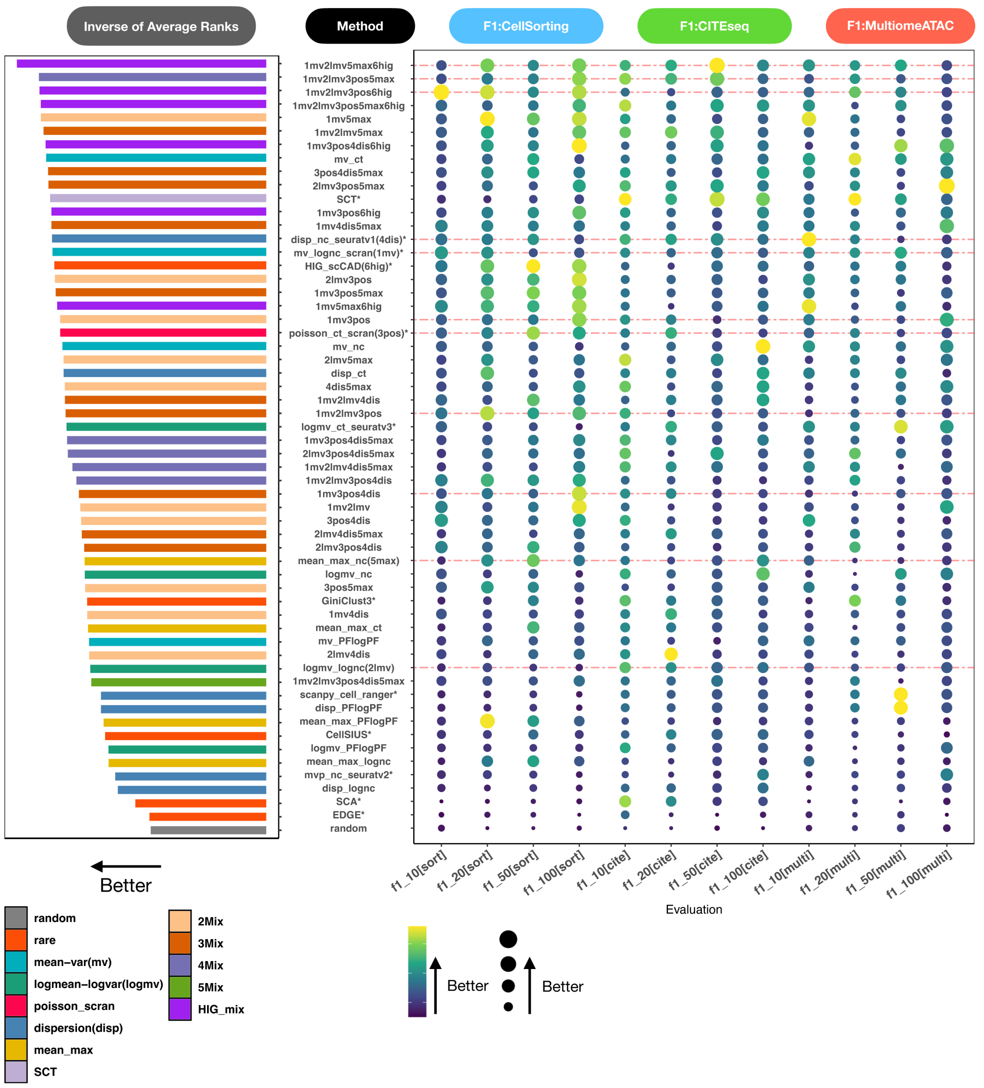


**Fig. S15**: Rare cell type identification performance using genes selected by baseline and hybrid HVG and RCTI methods, shown in descending order of overall performance. For each data type, evaluations were conducted at four rare cell type abundance levels with 10, 20, 50, or 100 cells, respectively. The balloon plots depict the average performance across all benchmark datasets for each method and abundance level, with methods ranked by the $F_1$ score. The bar plot on the left shows the average rank across all 12 evaluation settings. Hybrid HVG methods are color-coded by the number of baseline methods they incorporate, with each hybrid named according to its combination of baseline methods. Hybrids combining HVG and RCTI methods are shown in purple. In the balloon plot, the top three methods based on RCTI performance, the top three methods from the HVG benchmark in Figure 6 along with the baseline methods used to construct them are highlighted with red dashed lines.

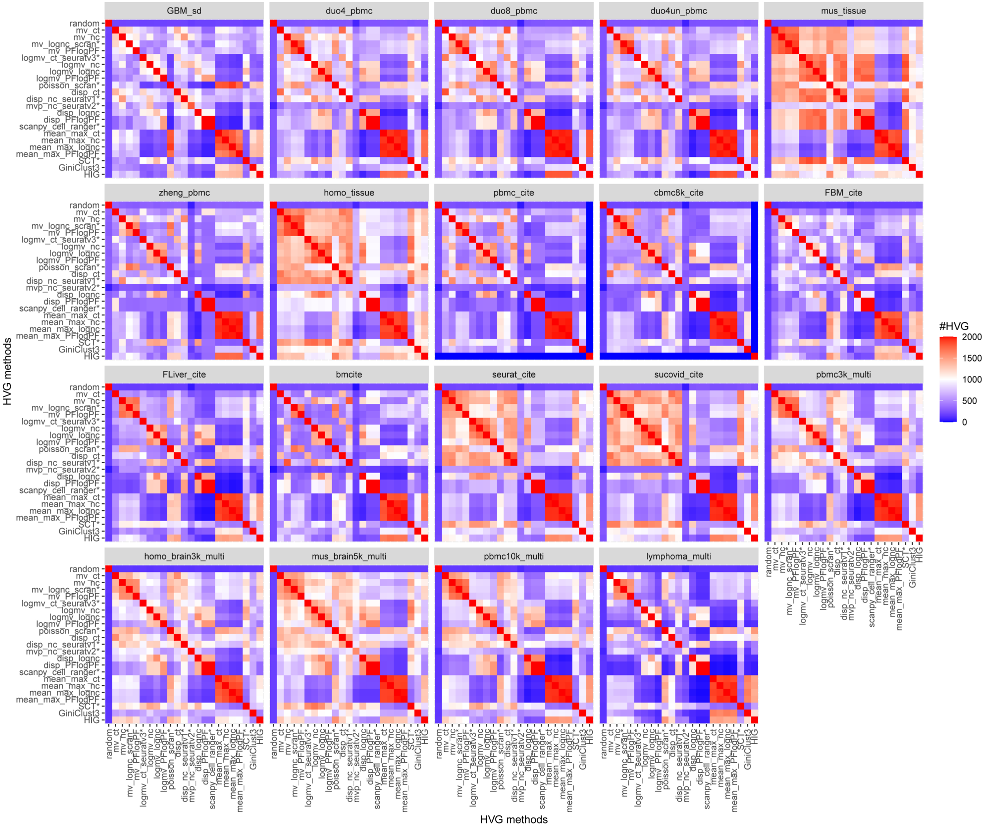


**Fig. S16**: Heatmap showing the number of overlapping genes among the top 2,000 genes selected by the baseline HVG and RCTI methods. Each subplot represents one dataset. Red indicates high overlap. For RCTI methods, only GiniClust3 and HIG_scCAD output gene lists and therefore are included.

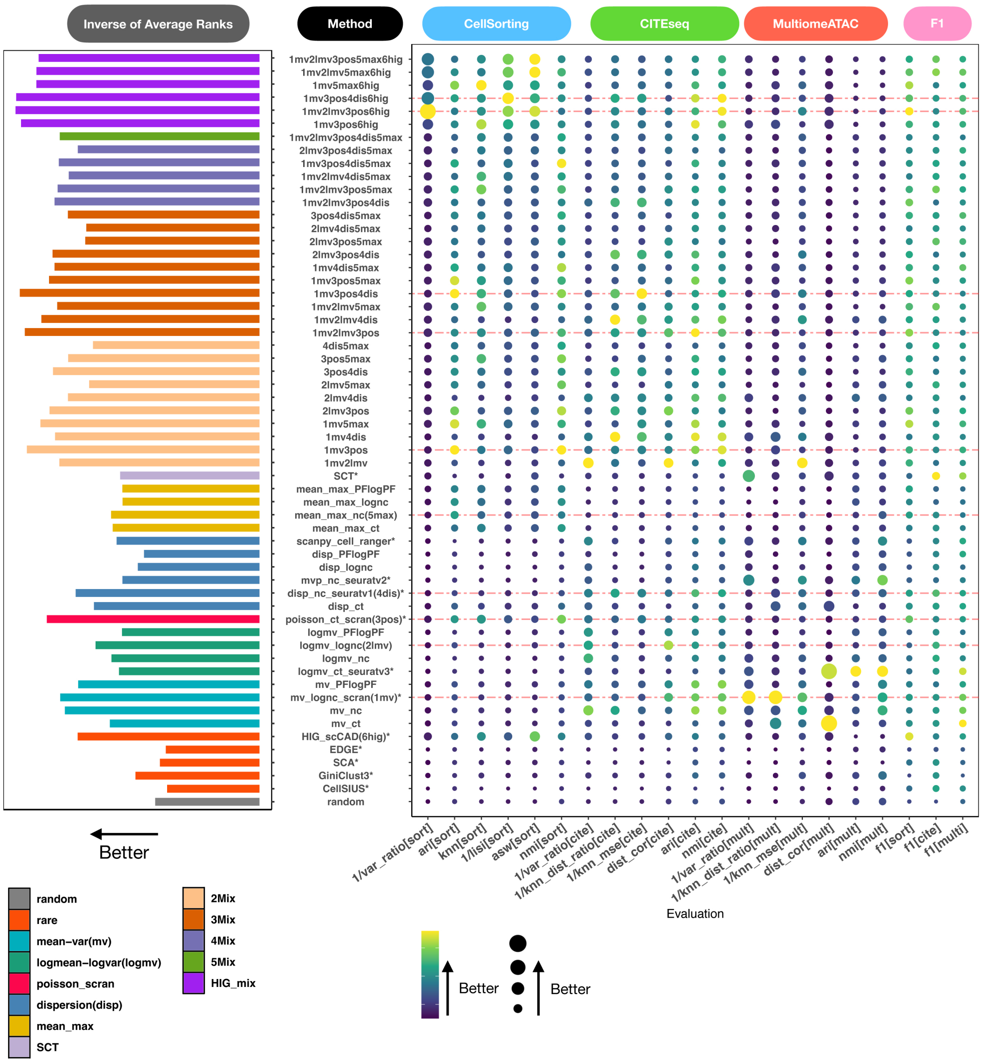


**Fig. S17**: Evaluation of HVG and RCTI methods across all benchmark datasets and criteria. The evaluation covers 21 criteria, including 18 for HVG and 3 for RCTI, grouped by benchmark data type (cell sorting, CITE-seq, and multiomeATAC). Average performance for each method and criterion is shown in a balloon plot, with methods ranked separately for each criterion. The bar plot on the left displays the average rank across all 21 criteria. Hybrid HVG methods are color-coded by the number of baseline methods they incorporate, and each hybrid is named according to the combination of baseline methods used. Hybrids combining HVG and RCTI methods are shown in purple. In the balloon plot, the top three methods based on overall performance, the top three methods from the HVG benchmark in Figure 6 along with the baseline methods used to construct them are highlighted with red dashed lines.

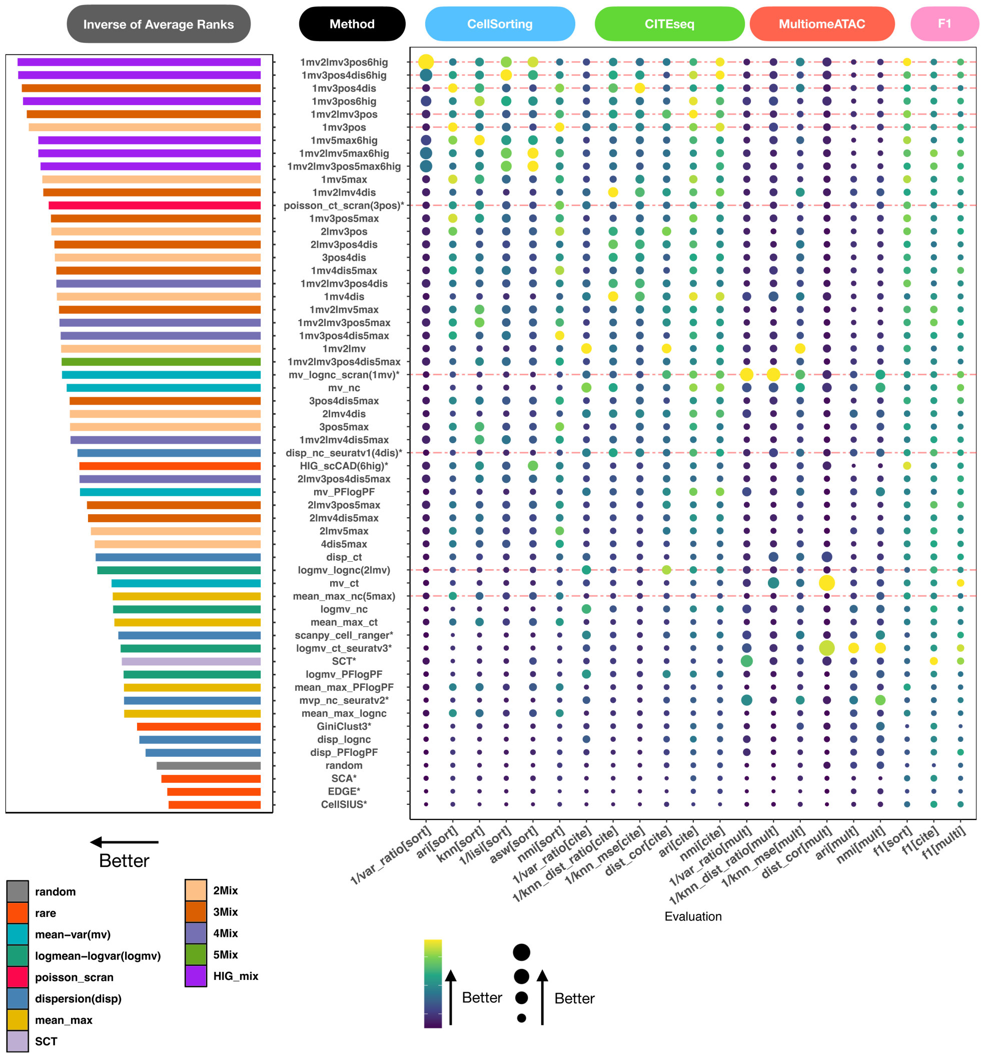


**Fig. S18**: Evaluation of HVG and RCTI methods across all benchmark datasets and criteria, with methods displayed in descending order of overall performance. The evaluation covers 21 criteria, including 18 for HVG and 3 for RCTI, grouped by benchmark data type (cell sorting, CITE-seq, and multiomeATAC). Average performance for each method and criterion is shown in a balloon plot, with methods ranked separately for each criterion. The bar plot on the left displays the average rank across all 21 criteria. Hybrid HVG methods are color-coded by the number of baseline methods they incorporate, and each hybrid is named according to the combination of baseline methods used. Hybrids combining HVG and RCTI methods are shown in purple. In the balloon plot, the top three methods based on overall performance, the top three methods from the HVG benchmark in Figure 6 along with the baseline methods used to construct them are highlighted with red dashed lines.

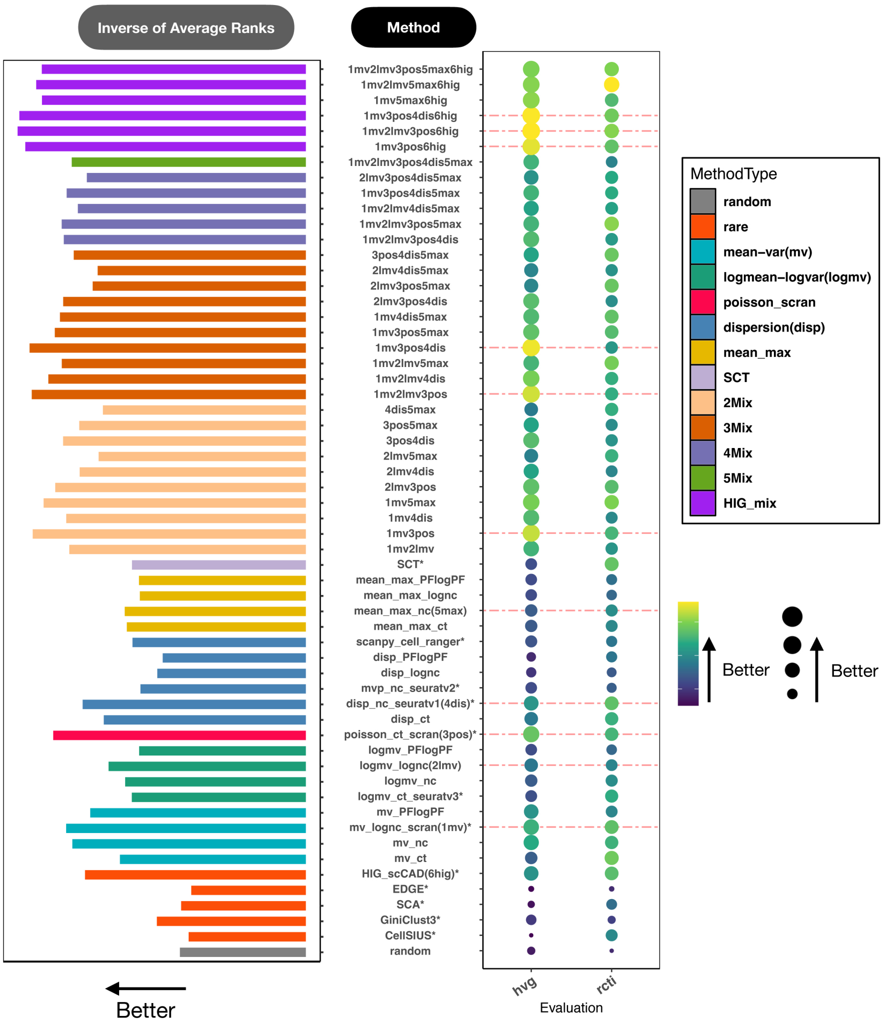


**Fig. S19**: Evaluation of HVG and RCTI methods across all benchmark datasets and criteria, with HVG and RCTI criteria equally weighted. For each method, the average rank across the 18 HVG evaluation criteria and the average rank across the 3 RCTI criteria were first computed and shown in a balloon plot. These two averages were then averaged to derive the final performance rank for each method. The bar plot on the left displays ranks based on this final performance. Hybrid HVG methods are color-coded by the number of baseline methods they incorporate, with each hybrid named according to its combination of baseline methods. Hybrids combining HVG and RCTI methods are shown in purple. In the balloon plot, the top three methods based on overall performance, the top three methods from the HVG benchmark in Figure 6 along with the baseline methods used to construct them are highlighted with red dashed lines.

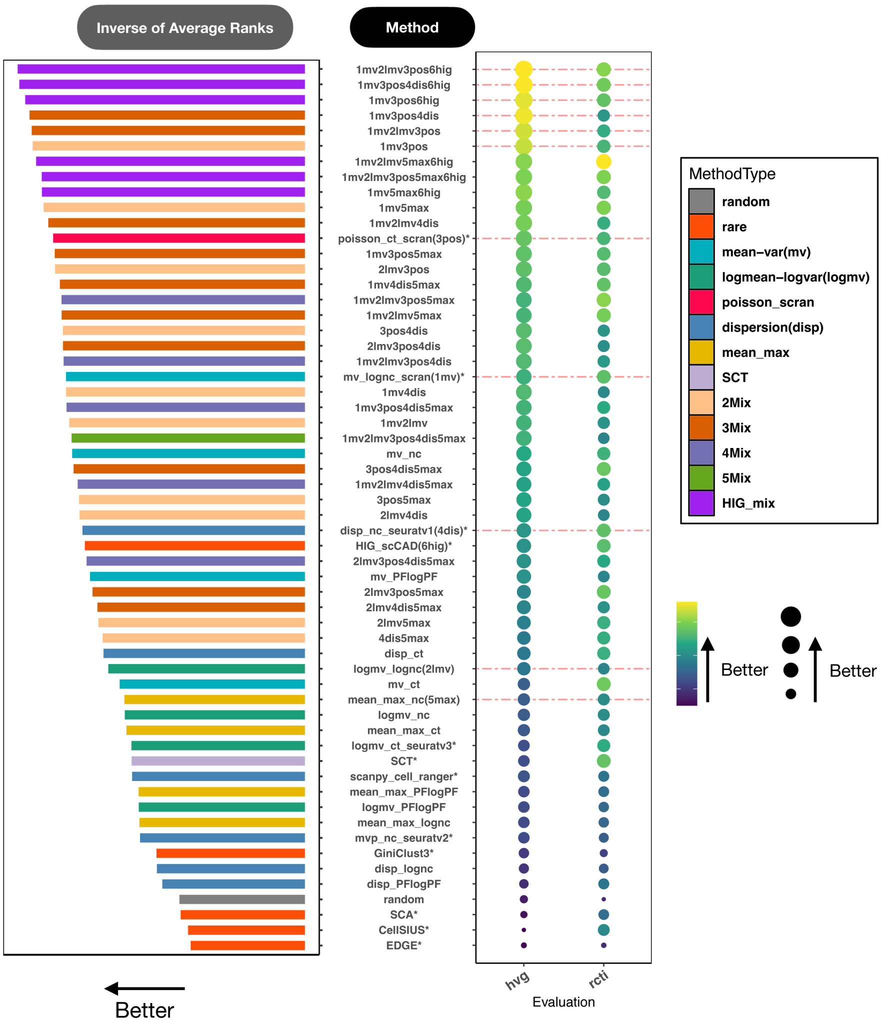


**Fig. S20**: Evaluation of HVG and RCTI methods across all benchmark datasets and criteria, with HVG and RCTI criteria equally weighted and methods displayed in descending order of overall performance. For each method, the average rank across the 18 HVG evaluation criteria and the average rank across the 3 RCTI criteria were first computed and shown in a balloon plot. These two averages were then averaged to derive the final performance rank for each method. The bar plot on the left displays ranks based on this final performance. Hybrid HVG methods are color-coded by the number of baseline methods they incorporate, with each hybrid named according to its combination of baseline methods. Hybrids combining HVG and RCTI methods are shown in purple. In the balloon plot, the top three methods based on overall performance, the top three methods from the HVG benchmark in Figure 6 along with the baseline methods used to construct them are highlighted with red dashed lines.

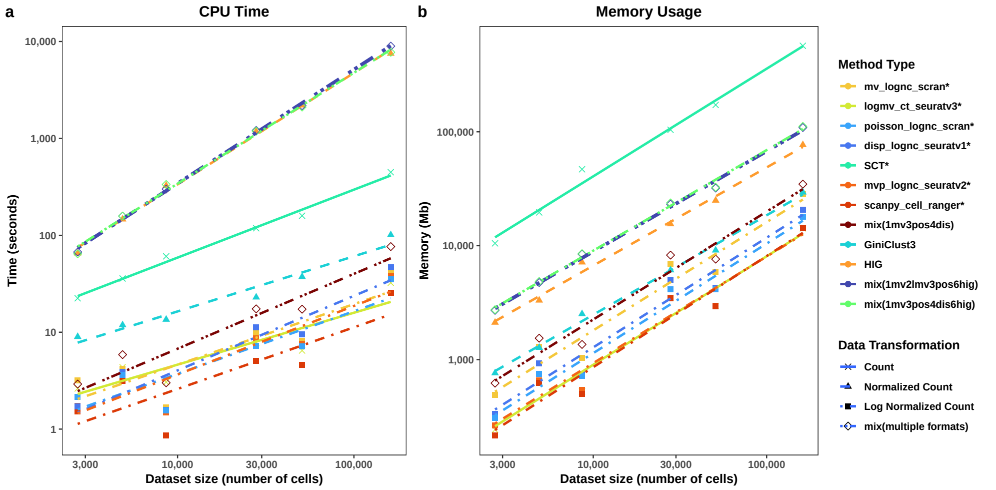


**Fig. S21**: Computational efficiency and scalability of gene selection by HVG and RCTI methods. **(a)** CPU time and **(b)** memory usage are shown for datasets of varying sizes (number of cells). The comparison includes seven existing HVG methods with publicly available software tools (denoted by an asterisk *), two RCTI methods (GiniClust3 and HIG_scCAD) capable of outputting selected genes, and the optimal hybrid methods based on overall HVG and RCTI performance (1mv2lmv3pos6hig, 1mv3pos4dis6hig, and 1mv3pos4dis). Among these, 1mv3pos4dis is also the top-performing method in the HVG-only benchmark (Figure 6). The datasets used for this analysis include pbmc3k_multi, mus_brain5k_multi, cbmc8k_cite, FLiver_cite, mus_tissue, seurat_cite. They are arranged based on increasing cell numbers.

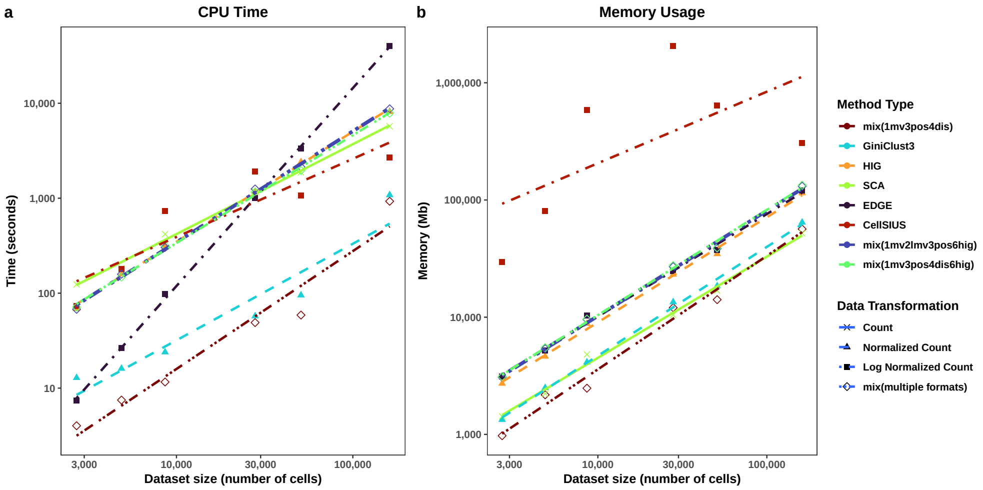


**Fig. S22**: Computational efficiency and scalability of RCTI methods and optimal hybrid methods for end-to-end analysis, from input expression matrix to final cell clusters. **(a)** CPU time and **(b)** memory usage are shown for datasets of varying sizes (number of cells). The comparison includes five existing RCTI methods with publicly available software tools (GiniClust3, HIG_scCAD, SCA, EDGE, and CellSIUS) and the optimal hybrid methods based on overall HVG and RCTI performance (1mv2lmv3pos6hig, 1mv3pos4dis6hig, and 1mv3pos4dis). Among these, 1mv3pos4dis is also the top-performing method in the HVG-only benchmark (Figure 6). The datasets used for this analysis include pbmc3k_multi, mus_brain5k_multi, cbmc8k_cite, FLiver_cite, mus_tissue, seurat_cite. They are arranged based on increasing cell numbers.
