# Supplementary material for: A systematic evaluation of highly variable gene selection methods for single-cell RNA-sequencing
Source: Genome Biol. 2025 Dec 11;26:424. doi: 10.1186/s13059-025-03887-x (PMC12699822; doi:10.1186/s13059-025-03887-x)
Supplement: Supplementary file 2 — Additional file 2: Supplementary Results and Methods [file 13059_2025_3887_MOESM2_ESM.pdf]

Additional File 2: Supplementary Results and Methods

Supplementary Results

S1. Further Exploration of Data Type–Dependent Patterns in Method Performance

Fig. 5a reveals a data type-dependent pattern in the performance of HVG selection methods. Some methods, particularly those based on the mean_max approach, perform well in the cell sorting benchmark data but less effectively in the CITE-seq and multiomeATAC datasets. Conversely, other methods, such as mean-var-based methods, ranked lower in the cell sorting data but showed better performance in CITE-seq and multiomeATAC data. To better understand this discrepancy, we conducted additional analyses.

To begin, we used orthogonal information to define cell types for each dataset (i.e., cell sorting labels, or clusters derived from ADT or ATAC-seq data). We then identified cell type-specific marker genes using Seurat's FindAllMarkers function. The number of cell types in the cell sorting datasets was determined based on the available cell type labels. For the CITE-seq and multiomeATAC datasets, the number of cell types was determined as follows. We first performed Louvain clustering across a range of resolutions from 0.1 to 2, in increments of 0.1, separately for the scRNA-seq data and the ADT or ATAC data. For each pair of resolutions, we computed ARI and NMI, and recorded the resolution pairs that yielded the optimal ARI and NMI for each combination of dataset, HVG selection method, and evaluation criterion (ARI or NMI). For each dataset, we then selected the resolution pair at which most methods performed best, and used the corresponding resolution for ADT or ATAC to generate the cell clustering. This clustering defined both the cell types and the number of cell types. For each cell type, marker genes were defined as those with significantly upregulated expression compared to all other cells, based on the Wilcoxon rank-sum test (Bonferroni adjusted p-value ≤ 0.05, log2 fold change ≥ 0.25). The union of marker genes across all cell types was treated as the gold standard marker gene set, which reflects transcriptomic differences across cell types. Importantly, this marker set was derived from cell types identified through orthogonal information, rather than from scRNA-seq data.

Next, we examined whether the gold standard marker genes tend to have higher expression levels in the cell sorting datasets, since mean_max methods, which select

genes with the highest expression, performed better in the cell sorting datasets. For each dataset, we computed the proportion of gold standard marker genes that are highly expressed, defined as the top 2000 genes with the highest mean expression levels. Although not statistically significant, this proportion showed a higher mean in the cell sorting datasets compared to the CITE-seq and multiomeATAC data (Additional file 1: Fig. S9a), suggesting that marker genes in cell sorting datasets tend to have high expression levels. These findings suggest that mean_max methods may more effectively identify cell type markers in cell sorting datasets, but not necessarily in the other data types.

To further investigate this hypothesis, we evaluated each HVG selection method's ability to recover cell type marker genes. Specifically, for each dataset, we computed the proportion of the top k HVGs identified by each method that overlapped with the gold standard marker gene set. Additional file 1: Fig. S9b summarizes the results, showing average performance across datasets within each benchmark data type. In cell sorting data, mean-max-based methods and poisson_scran performed similarly, both outperforming others in recovering marker genes. However, in the CITE-seq and multiomeATAC datasets, some other methods surpassed the mean_max approaches in this regard. This performance pattern helps explain the observed variability in method effectiveness across different data types.

Since method performance is ranked for each evaluation criterion, a method that performs well receives a higher rank and a larger balloon in the balloon plots, while lower-performing methods receive smaller balloons. Thus, mean_max methods show large balloons in the cell sorting datasets, while other methods appear smaller. In contrast, the reverse is observed in CITE-seq and multiomeATAC datasets, where other methods yield larger balloons. Notably, some methods such as poisson_scran (3pos) consistently perform well across all three data types in recovering cell type markers and accordingly receive strong ranks and medium to large balloons across the evaluation criteria.

S2. Factors Influencing the Performance of Hybrid Methods

Fig. 6 shows that certain hybrid methods performed better than the others. We investigated this further and found that a hybrid method's performance may be influenced by both the diversity of its selected HVGs and the performance of its constituent baseline methods.

To explore this, we plotted the average rank performance of hybrid methods and the five baseline methods used to construct them, stratified by the number of baseline methods included. We transformed the ranks using the inverse (max_rank/r, where r is a method's mean rank across all evaluation criteria and max_rank is the maximum r among all methods), so that larger values indicate better performance. Performance initially improved as more baseline methods were added, but declined or plateaued when four or more were included (Additional file 1: Fig. S13a).

This decline appears to reflect the quality of the constituent baseline methods. For example, the baseline method mean_max_nc (5max) selects genes with little overlap with those from many other methods (Fig. 5b, Additional file 1: Fig. S11), increasing

diversity. However, because 5max generally performs worse than other baseline methods (e.g., 1mv, 2lmv, 3pos, 4dis), its inclusion often reduces hybrid performance. For instance, 1mv2lmv4dis outperforms 1mv2lmv4dis5max. Additional file 1: Fig. S13b compares hybrid method pairs with and without 5max, revealing a drop in performance when 5max is included for most pairs. Notably, 4 of the 5 hybrids with four constituent methods (4mix hybrid) contain 5max, and the only hybrid with five constituent methods (5mix hybrid) also includes it. This explains why hybrid performance plateaued or declined once the number of constituent baseline methods reached four (Additional file 1: Fig. S13a).

We quantified this relationship by correlating each hybrid method's performance with the average performance of its constituent baseline methods, finding a strong correlation (Additional file 1: Fig. S13c; Pearson correlation = 0.758).

To disentangle the effects of HVG diversity and baseline quality, we fit a multiple regression model with overall performance rank as the response and two predictors: (1) the average performance of constituent baseline methods, and (2) HVG diversity, defined as the number of baseline methods used. As above, ranks were transformed so that larger values indicate better performance. The results are summarized in Additional file 3: Table S1. When the model was fit using all hybrid methods and the five baseline methods used to construct hybrids, average baseline performance was significantly associated with better ranks (two-sided p-value = 4.3×10^{-7}), while diversity showed a positive but non-significant effect (two-sided p-value = 0.067). However, when the model was fit after excluding all methods involving 5max, both predictors became significant (two-sided p-value = 0.00047 for baseline performance and 0.0029 for diversity).

We also tested an alternative diversity metric based on Shannon entropy. Here, we first identified method-specific genes among the top 2000 HVGs, defined as genes selected only by one constituent baseline method. We computed the proportion of method-specific genes from each baseline method, p_i. Entropy was then calculated as $-\sum_i p_i \log_2(p_i)$, with larger values indicating greater diversity. Entropy correlates with performance rank (Additional file 1: Fig. S13d; Pearson correlation = 0.256). In a multiple regression including both entropy and average baseline performance, both predictors were significant (two-sided p-value = 6.2×10^{-8} and 0.0059, respectively).

Overall, these findings indicate that strong hybrid performance depends on balancing HVG diversity from complementary methods with high individual performance of the baseline methods included.

S3. Feature Selection for Rare Cell Type Identification

Although this study primarily focuses on evaluating methods for selecting globally varying features, we also compared 47 HVG methods with six state-of-the-art methods designed for rare cell type identification (RCTI), including scCAD [1], GiniClust3 [2], EDGE [3], SCA [4], GapClust [5], and CellSIUS [6]. These RCTI methods were selected based on their strong performance or widespread use in prior studies [1].

Among these RCTI methods, GapClust relies on Seurat v1 for feature selection, so its gene selection performance is not presented separately, as it mirrors that of disp_nc_seuratv1 (4dis). SCA and EDGE do not output selected genes; instead, they

generate cell embeddings, which we directly used for clustering cells with the Louvain algorithm and for downstream evaluation. In contrast, scCAD and GiniClust3 do output gene features. scCAD uses Seurat v1 for HVG selection and employs a highly important gene (HIG) approach for rare cell type-associated feature identification. We focused on the HIGs (HIG_scCAD) for evaluation, as Seurat v1 is already benchmarked. GiniClust3 selects genes using a normalized Gini index. For both scCAD and GiniClust3, we used the top 2,000 genes to ensure consistency with other methods. CellSIUS outputs both cell-level labels indicating whether each cell belongs to a rare cell type and a set of marker genes associated with those rare cell types. However, the number of marker genes is typically much smaller than 2,000, with an average of 182 genes per dataset in our benchmark. Therefore, we used the rare cell type labels directly to evaluate CellSIUS's performance in rare cell type detection. For assessing its ability to capture global variation using the 18 HVG evaluation criteria, we used its output marker genes as-is.

To assess rare cell type detection, we generated benchmark data using the 19 datasets in Table 1 as templates. For each dataset, ground-truth cell types were defined based on orthogonal information (e.g., cell sorting, ADTs in CITE-seq, or ATAC LSIs in multiome data). We then randomly selected one cell type and downsampled it to create rare populations of 10, 20, 50, or 100 cells, resulting in $19 \times 4 = 76$ datasets with known rare cell type labels.

After feature selection with each method, we clustered the scRNA-seq data and compared the resulting clusters to the ground-truth rare cell type labels. RCTI performance was evaluated using the F_1 score, a widely used metric for rare cell type identification [1, 4, 5]. If a method successfully identifies the rare cell type, one of its scRNA-seq derived clusters should closely match the rare cell type. Thus, for each method, we computed the F_1 score for each cluster and reported the maximum score as the method's final F_1 value. Methods were then ranked based on their maximum F_1 scores and the rankings were averaged across all datasets.

Among the five RCTI methods evaluated (excluding GapClust as it uses Seurat v1), HIG_scCAD achieved the best overall performance in identifying rare cell types (Additional file 1: Figs. S14–S15). However, several baseline HVG selection methods, such as mv_ct, disp_nc_seuratv1 (4dis), mv_lognc_scran (1mv), and SCT, performed comparably. Notably, 4dis is used by both Seurat v1 and GapClust. In addition, several hybrid HVG methods matched or exceeded the performance of HIG_scCAD. The top-performing hybrid methods — 1mv2lmv3pos5max, 1mv5max, and 1mv2lmv5max — all included the baseline method mean_max_nc (5max). Notably, the genes selected by the top-performing RCTI method, HIG_scCAD, showed greater overlap with those identified by mean-max-based HVG methods than with those identified by other HVG methods (Additional file 1: Fig. S16).

To further evaluate the impact of combining RCTI and HVG approaches, we created six new hybrid methods by integrating HIG_scCAD (6hig) with the top-performing HVG methods from Fig. 6 and Additional file 1: Fig. S15: 1mv3pos4dis6hig, 1mv2lmv3pos6hig, 1mv3pos6hig, 1mv2lmv3pos5max6hig, 1mv5max6hig, and 1mv2lmv5max6hig (Additional file 2: Supplementary Methods). These hybrid methods showed comparable or improved RCTI performance relative to

HIG_scCAD, with 1mv2lmv5max6hig achieving the best performance (Additional file 1: Figs. S14–S15).

We also evaluated the ability of the RCTI methods to identify globally variable genes using our 18 HVG evaluation criteria. The baseline RCTI methods did not demonstrate competitive performance in this task (Additional file 1: Figs. S17-S18). We then compared all methods across 21 evaluation criteria: 18 HVG metrics and 3 aggregated RCTI metrics (reflecting average F_1 score rankings across the three benchmark data types). Overall, hybrid methods outperformed baseline methods, including the RCTI methods. The two best-performing hybrid methods — 1mv2lmv3pos6hig and 1mv3pos4dis6hig — combine the top HVG methods with HIG_scCAD. The top HVG method from Fig. 6, 1mv3pos4dis, was also the third-best method overall when RCTI performance was included. When we applied equal weights to HVG and RCTI metrics and averaged ranks accordingly (Additional file 1: Figs. S19–S20), similar conclusions were reached, with 1mv2lmv3pos6hig, 1mv3pos4dis6hig, and 1mv3pos6hig ranked in the top three, followed by 1mv3pos4dis.

These results suggest that incorporating RCTI-specific feature selection strategies can further enhance the performance of hybrid HVG methods, making them highly effective for both global and rare cell type–targeted feature selection. While our study included several leading RCTI methods, a comprehensive benchmark of all existing RCTI approaches is beyond the current scope. Nonetheless, our findings demonstrate that hybrid methods can outperform baseline methods in identifying both globally variable features and features associated with rare cell types.

Finally, we assessed the computational efficiency and scalability of the RCTI methods and two top-performing hybrid methods (1mv2lmv3pos6hig and 1mv3pos4dis6hig). For HIG_scCAD, GiniClust3, and the hybrid methods, which all output a ranked list of genes, we evaluated their resource usage during feature selection (Additional file 1: Fig. S21). For these methods, both runtime and memory usage scaled linearly with cell number on the log scale. HIG_scCAD and its associated hybrids were the slowest and most memory-intensive, exceeding all other HVG methods except SCT. GiniClust3 was more efficient than HIG_scCAD but still slower and more memory-intensive than most HVG methods. For SCA, EDGE, and CellSIUS, which do not output a ranked gene list, we evaluated end-to-end resource usage (from input expression matrix to final cell clusters; Additional file 1: Fig. S22). These methods were also slower than GiniClust3. CellSIUS consumed the most memory, while EDGE and the HIG_scCAD-based hybrids had similar high memory requirements. SCA's memory usage was comparable to GiniClust3. In summary, while 1mv2lmv3pos6hig and 1mv3pos4dis6hig achieved the best gene selection performance, they were computationally slower and less memory efficient. In contrast, 1mv3pos4dis, which ranked third in overall performance, was significantly more efficient in terms of both runtime and memory.

Supplementary Methods

Rare Cell Type Feature Selection Methods

GiniClust3

GiniClust3 [2] employs the Gini index, a metric originally developed to measure social inequality, to identify genes with highly skewed expression patterns. For each gene, cells are ranked by expression level, and the cumulative expression is plotted as a Lorenz curve. The Gini index is computed as twice the area between the Lorenz curve and the diagonal line of equality. It has been observed that Gini index values are strongly correlated with genes' maximum expression levels. To account for this, a two-step LOESS regression procedure is used to normalize the Gini index values. First, a LOESS curve is fitted to regress the Gini values against log-transformed maximum expression levels, and genes with large residuals (outliers) are removed. A second LOESS fit is then applied to the remaining genes. The normalized Gini index for each gene is calculated by subtracting the fitted value from its original Gini score. P-values are estimated via normal approximation, and a p-value threshold is applied to select exactly 2,000 genes for downstream analysis, ensuring fair comparison with other methods.

SCA

Surprisal Component Analysis (SCA) [4] begins by computing a surprisal score for each gene in each cell using the Wilcoxon rank-sum test. This score quantifies how much a gene's expression in a cell's k-nearest-neighbor (kNN) neighborhood deviates from its global expression pattern. This results in a matrix of surprisal scores, where rows represent cells and columns represent genes. The right eigenvectors of the matrix serve as loading vectors, representing linear combinations of genes that capture the principal variations in the surprisal matrix. A low-dimensional embedding is obtained by projecting the original expression data onto these loading vectors. This procedure can be iteratively refined: the embedding is used to recompute cell neighborhoods, which in turn are used to construct a new surprisal matrix, followed by another round of decomposition to generate an updated embedding. In our analysis, we use the final embedding output by SCA for all downstream tasks. According to the original authors, traditional dimensionality reduction methods, such as PCA, optimize global objectives that may overlook rare or subtly defined cell populations. For example, PCA captures features that explain total variance across the dataset, potentially diluting signals from small or local subpopulations. In contrast, SCA emphasizes local variation by computing surprisal scores at the single-cell and gene level within cell neighborhoods, making it better suited for preserving information about rare or nuanced cell types.

EDGE

Ensemble Dimensionality Reduction and Feature Gene Extraction (EDGE) [3] is designed to simultaneously produce a low-dimensional embedding and identify informative feature genes. At its core, EDGE constructs an ensemble of "weak learners", each built from a random subset of genes and partitioning cells into distinct groups, referred to as hash codes. These weak learners serve two main purposes: collectively, they vote on cell-to-cell similarities; individually, they contribute to assessing the importance of the genes involved in their construction. Although signals from rare

cell types are often subtle and easily masked by dominant populations, random gene subsets used in individual weak learners offer diverse "views" of the data, enabling the ensemble to capture these rare signals more effectively. This ensemble approach also enhances robustness to noise. To generate the embedding, similarity votes from all weak learners are averaged to form a consensus cell-to-cell similarity matrix. This matrix is then subjected to spectral embedding, followed by optimization, to obtain the final low-dimensional representation. Since the EDGE package only outputs the low-dimensional embedding, we used this embedding for all downstream analyses in our study.

scCAD

Cluster Decomposition-based Anomaly Detection (scCAD) [1] employs Seurat v1 to identify the top 2,000 Highly Variable Genes (HVGs). In parallel, it selects 2,000 Highly Important Genes (HIGs) for distinguishing cell types, including rare cell types. To identify HIGs, scCAD performs an initial clustering using the Louvain algorithm. It then trains a random forest classifier with the gene expression matrix as input and the cluster labels from the initial clustering as targets. Each gene is assigned an importance score based on its contribution to the classification, and the top-ranked genes are selected as HIGs. The feature selection followed by iterative cluster decomposition and merging enables scCAD to identify rare cell types.

GapClust

GapClust [5] utilizes Seurat v1 for feature selection, relying on its highly variable gene selection pipeline as a preprocessing step.

CellSIUS

Cell Subtype Identification from Upregulated Gene Sets (CellSIUS) [6] operates on a pre-defined initial clustering of cells and identifies sub-clusters within each cluster to detect rare subpopulations. In this study, the initial clustering was generated using the scran method (mv_lognc_scran). For each initial cluster, CellSIUS first identifies candidate marker genes with bimodal expression distributions, determined using one-dimensional k-means clustering. For each candidate gene, the mean expression of the higher mode within the cluster is compared with the mean expression of the same gene in cells outside the cluster, and only those significantly upregulated in the higher mode are retained. These retained cluster-specific markers are then grouped into correlated gene sets via a graph-based clustering algorithm applied to a gene–gene correlation network. The resulting gene sets serve as signatures to identify rare cell subtypes within each cluster. The output of CellSIUS includes both (i) cell-level labels indicating whether each cell belongs to a rare cell type and (ii) marker genes associated with those rare cell types. In our benchmark datasets, CellSIUS reported an average of 182 marker genes per dataset. Since this number is fewer than 2,000, we used the reported rare cell type labels directly to evaluate rare cell type identification performance. To assess its ability to capture global variation using the 18 HVG evaluation criteria, we used the reported marker genes as-is.

Hybrid HVG and RCTI Methods

The hybrid methods that incorporate both HVG and RCTI baseline methods were constructed following the same procedure described in the main manuscript.

RCTI Method Nomenclature

For baseline RCTI methods, we used HIG_scCAD to denote the highly important genes identified by scCAD. The other RCTI methods were labeled using their original names: EDGE, SCA, GiniClust3, CellSIUS, and GapClust.

For hybrid methods, we used 6hig as the abbreviation for HIG_scCAD. Each hybrid method was named by concatenating the abbreviations of its constituent baseline methods. For example, a hybrid method combining mv_lognc_scran, poisson_scran, disp_nc_seuratv1, and HIG_scCAD is denoted as 1mv3pos4dis6hig.

RCTI Benchmark Data and Processing

For each of the 19 datasets in Table 1, a randomly selected cell type was downsampled to create rare populations of 10, 20, 50, or 100 cells, resulting in $19 \times 4 = 76$ datasets with known rare cell type labels.

Given the selected genes, we processed the data using the same downstream analysis procedure as before to generate cell embeddings, compute cell distances, and perform cell clustering. If a method outputs only cell embeddings, we used those embeddings for the downstream analyses, including computing cell distances and performing cell clustering.

Gene Overlap Analysis for RCTI Methods

To compare the genes selected by RCTI and HVG methods, we first calculated the number of overlapping genes between each pair of methods within each benchmark dataset. For each of the 19 original datasets listed in Table 1, we generated four versions by varying the number of cells in the rare cell type. The gene overlap counts were averaged across these four versions for each dataset and are summarized in Additional file 1: Fig. S16.

Evaluation Criteria

Criteria for Rare Cell Type Identification

F_1 score: The F_1 score is the harmonic mean of two key metrics: precision and recall. Precision is the proportion of cells correctly identified as members of rare cell types out of all cells predicted to belong to rare cell types. Recall is the proportion of cells correctly identified as members of rare cell types out of all actual rare cell type members. The F_1 score provides a balanced measure that accounts for both false positives and false negatives, making it especially useful in evaluating performance on imbalanced datasets such as those involving rare cell types.

Overall Performance Ranking of Methods

To compare methods based on their overall performance in identifying both globally variable genes and rare cell types, we used a total of 21 evaluation criteria, comprising 18 HVG metrics and 3 aggregated RCTI metrics (the latter reflecting average F_1 score rankings across the three benchmark data types). Each method was ranked according to its performance on each individual metric, and the average rank across all 21 metrics was used to generate the final overall ranking, as shown in Additional file 1: Figs. S17–S18.

Because the number of HVG metrics exceeded that of RCTI metrics (18 vs. 3), we also applied an alternative ranking strategy that assigned equal weight to HVG and RCTI performance. In this approach, we first computed an average rank for each method across the 18 HVG metrics to obtain a ranking for global variation. Separately, we computed the average rank across the 3 RCTI metrics to obtain a ranking for rare cell type identification. We then averaged each method's HVG and RCTI ranks to produce a balanced overall ranking, presented in Additional file 1: Figs. S19–S20.

References

[1] Xu Y, Wang S, Feng Q, Xia J, Li Y, Li HD, et al. scCAD: Cluster decomposition-based anomaly detection for rare cell identification in single-cell expression data. Nature Communications. 2024;15:7561.

[2] Dong R, Yuan GC. GiniClust3: a fast and memory-efficient tool for rare cell type identification. BMC Bioinformatics. 2020;21:158.

[3] Sun X, Liu Y, An L. Ensemble dimensionality reduction and feature gene extraction for single-cell RNA-seq data. Nature Communications. 2020;11:5853.

[4] DeMeo B, Berger B. SCA: recovering single-cell heterogeneity through information-based dimensionality reduction. Genome Biology. 2023;24:195.

[5] Fa B, Wei T, Zhou Y, Johnston L, Yuan X, Ma Y, et al. GapClust is a light-weight approach distinguishing rare cells from voluminous single cell expression profiles. Nature Communications. 2021;12:4197.

[6] Wegmann R, Neri M, Schuierer S, Bilican B, Hartkopf H, Nigsch F, et al. CellSIUS provides sensitive and specific detection of rare cell populations from complex single-cell RNA-seq data. Genome Biology. 2019;20:142.
