# Supplementary material for: A systematic evaluation of highly variable gene selection methods for single-cell RNA-sequencing
Source: Genome Biol. 2025 Dec 11;26:424. doi: 10.1186/s13059-025-03887-x (PMC12699822; doi:10.1186/s13059-025-03887-x)
Supplement: Supplementary file 3 — Additional file 3: Supplementary Tables. Tables S1-S3 [file 13059_2025_3887_MOESM3_ESM.pdf]

Additional File 3: Supplementary Tables

Table S1: Dependence of hybrid method performance on HVG diversity and mean baseline method performance, evaluated using multiple regression conducted with and without methods that include 5max.

Model fitting data	Covariate	Coefficient	SE	t statistic	p-value	95% C.I.
Including 5max	Baseline rank	1.1531	0.1763	6.540	4.34×10^{-7}	(0.7919, 1.5143)
	HVG diversity	0.0460	0.0241	1.909	0.0666	(-0.0034, 0.0954)
Excluding 5max	Baseline rank	1.0795	0.2272	4.751	0.000471	(0.5844, 1.5746)
	HVG diversity	0.1247	0.0335	3.722	0.002917	(0.0517, 0.1977)
Including 5max	Baseline rank	1.1937	0.1639	7.285	6.24×10^{-8}	(0.8581, 1.5293)
	Entropy	0.1618	0.0542	2.983	0.00585	(0.0507, 0.2729)

This table shows regression coefficients, standard errors, t-statistics, two-sided p-values, and 95% confidence intervals for different model configurations. The first two blocks compare models fitted with and without 5max methods using the number of constituent baseline methods as the HVG diversity metric, while the last block shows results using Shannon's entropy as the HVG diversity metric.

Table S2: Criteria for filtering low quality cells in scRNA-seq.

Dataset	Filtering criteria
GBM_sd	Keep cells with $8 \times 10^4 \leq$ nCount_RNA $\leq 9 \times 10^5$
mus_tissue	Keep cells with nFeature_RNA ≥ 1000 and genes expressed in $\geq 0.1\%$ of cells
zheng_pbmc	Keep cells with nFeature_RNA ≥ 1000 and genes expressed in $\geq 0.1\%$ of cells
homo_tissue	Keep cells with nFeature_RNA ≥ 500 and genes expressed in $\geq 0.1\%$ of cells
pbmc_cite	Remove genes and cells associated with mouse
cbmc8k_cite	Remove genes and cells associated with mouse
FLiver_cite	Keep cells with both scRNA-seq and ADT
FBM_cite	Keep cells with both scRNA-seq and ADT
sucovid_cite	Follow the processing protocol in [1]
pbmc3k_multi	Keep cells with $1 \times 10^3 \leq$ nCount_ATAC $\leq 5 \times 10^4$ $1 \times 10^3 \leq$ nCount_RNA $\leq 2.5 \times 10^4$, percent.mt ≤ 20
homo_brain3k_multi	Keep cells with $1 \times 10^3 \leq$ nCount_ATAC $\leq 2 \times 10^5$ $8 \times 10^2 \leq$ nCount_RNA $\leq 5 \times 10^4$, percent.mt ≤ 3
mus_brain5k_multi	Keep cells with $1 \times 10^3 \leq$ nCount_ATAC $\leq 1 \times 10^5$ $1 \times 10^3 \leq$ Count_RNA $\leq 5 \times 10^4$
pbmc10k_multi	Keep cells with $5 \times 10^3 \leq$ nCount_ATAC $\leq 7 \times 10^4$ $1 \times 10^3 \leq$ nCount_RNA $\leq 2.5 \times 10^4$, percent.mt ≤ 20
lymphoma_multi	Keep cells with $5 \times 10^2 \leq$ nCount_ATAC $\leq 7 \times 10^4$ $3 \times 10^2 \leq$ nCount_RNA $\leq 2 \times 10^4$, percent.mt ≤ 3

Note: nFeature_RNA: number of genes detected per cell in scRNA-seq. nCount_RNA: number of UMIs per cell in scRNA-seq. percent.mt: mitochondrial percentage of a cell in scRNA-seq. nCount_ATAC: number of sequencing reads detected per cell in scATAC-seq.

Table S3: Summary of methods, package, version, and parameters

Methods	Packages	Functions	Hyperparameters
disp_nc_seuratv1	Seurat 4.4.0	FindVariableFeatures	selection.method="disp", loess.span=0.3, clip.max="auto", mean.function=FastExpMean, dispersion.function=FastLogVMR, num.bin=20, binning.method="equal_width", nfeatures=2000, mean.cutoff=c(0.1, 8), dispersion.cutoff=c(1, Inf)
mvp_nc_seuratv2	Seurat 4.4.0	FindVariableFeatures	selection.method="mvp", loess.span=0.3, clip.max="auto", mean.function=FastExpMean, dispersion.function=FastLogVMR, num.bin=20, binning.method="equal_width", nfeatures=2000, mean.cutoff=c(0.1, 8), dispersion.cutoff=c(1, Inf)
logmv_ct_seuratv3	Seurat 4.4.0	FindVariableFeatures	selection.method="vst", loess.span=0.3, clip.max="auto", mean.function=FastExpMean, dispersion.function=FastLogVMR, num.bin=20, binning.method="equal_width", nfeatures=2000, mean.cutoff=c(0.1, 8), dispersion.cutoff=c(1, Inf)
SCT	Seurat 4.4.0	SCTransform	reference.SCT.model=NULL, do.correct.umi=TRUE, ncells=5000, residual.features=NULL, variable.features.n=2000, variable.features.rv.th=1.3, vars.to.regress=NULL, do.scale=FALSE, do.center=TRUE, clip.range=c(-sqrt(x=ncol(x=object[[assay]])/30), sqrt(x=ncol(x=object[[assay]])/30)), conserve.memory=FALSE, return.only.var.genes=TRUE, seed.use=1448145
mv_lognc_scran	scran 1.30.0	modelGeneVar	block=NULL, design=NULL, subset.row=NULL, subset.fit=NULL, equiweight=TRUE, method="fisher", BPPARAM=SerialParam())
poisson_scran	scran 1.30.0	modelGeneVarByPoisson	size.factors=NULL, block=NULL, design=NULL, subset.row=NULL, npts=1000, dispersion=0, pseudo.count=1, equiweight=TRUE, method="fisher", BPPARAM=SerialParam())
scanpy_cell_ranger	scanpy	highly_variable_genes	n_top_genes=2000, min_disp=0.5, max_disp=inf, min_mean=0.0125, max_mean=3, span=0.3, n_bins=20, flavor='cell_ranger', subset=False, inplace=True, batch_key=None, check_values=True
GiniClust3	giniclust3 1.1.2	calGini	Instead of using the default cutoff for p-values, we select 2000 genes with the smallest p-values.
SCA	shannonca 0.0.9	reduce	n_comps=30, iters=5, nbhds=None, nbhd_size=15, metric='euclidean', model='wilcoxon', keep_scores=False, keep_loadings=False, keep_all_iters=False, verbose=False, n_tests='auto', seed=10, chunk_size=None
EDGE	EDGE 1.0	endr	n_wl=500, n_dm=15, n_neigs=20, n_comps=2, n_epos=200, seed=5489, alpha=1, gamma=1, a=NA, b=NA, spread=1, min_dist=0.1, negative_sample_rate=5, H=101107, opt=FALSE
scCAD	scCAD 1.0.0	scCAD	seed=2023
CellSIUS	CellSIUS 1.0.0	CellSIUS	in_n_cells=10, min_fc=2, corr_cutoff=NULL, iter=0, max_perc_cells=50, fc_between_cutoff=1

References

[1] Zhang B, Ji Z, Ji H. Tree-based Correlation Screen and Visualization for Exploring Phenotype-Cell Type Association in Multiple Sample Single-Cell RNA-Sequencing Experiments. bioRxiv. 2021;bioRxiv:2021.10.27.466024.
